# Biomarkers to Measure Treatment Effects in Alzheimer's Disease: What Should We Look for?

**DOI:** 10.4061/2011/598175

**Published:** 2011-10-18

**Authors:** Kenneth Rockwood

**Affiliations:** Division of Geriatric Medicine, Dalhousie University and Centre for Health Care of the Elderly, Capital District Health Authority, 1421-5955 Veterans Memorial Lane, Halifax, NS, Canada B3H 2E1

## Abstract

It is often surprisingly difficult to tell whether a treatment for Alzheimer's disease is effective. Biomarkers might offer the potential of a quantifiable objective measure of treatment effectiveness. This paper suggests several criteria by which biomarkers might be evaluated as outcomes measures. These include biological plausibility, statistical significance, dose dependence, convergence across measures, and replicability. If biomarkers can meet these criteria, then, pending regulatory approval, they may have a role in the evaluation of treatment effectiveness in Alzheimer's disease. If not, their usefulness may be in supplementing, but not supplanting, clinical profiles of treatment effects.

For a compound to be a demonstrably effective treatment for Alzheimer's disease two broad conditions must be met: first, the compound must be effective, and, second, it must be tested in a way which allows that effectiveness to be demonstrated. As formidably difficult as the first challenge is, it also can be surprisingly tricky to show that any treatment for Alzheimer's disease that falls short of cure offers therapeutic potential. Even the tried-and-true Alzheimer's Disease Assessment Scale—Cognitive Subscale (ADAS-Cog) misclassifies important clinical change—typically overestimating decline [1]—so that more than twenty years into the modern era in dementia therapeutics, a recent consensus report has called for a new multidimensional measure for use in dementia drug trials [2].

Into this breech biomarkers seem poised to step. In general, a biomarker for dementia is the term given to “measurable biological characteristics that can either serve as indicators of normal or pathogenic processes in the body, or as tools to track pharmacological responses to therapeutic drugs” [3]. As the accompanying papers in this issue amply demonstrate, there are a host of sometimes ingenious measures that might be employed as biomarkers. Of particular interest is that some such measures might be detectable years before clinical dementia is present; in this way, they serve as targets for therapy, such that with successful treatment the manifestations of Alzheimer's disease are either attenuated or even absent. The challenges to achieving this heroic goal are formidable, requiring an advanced understanding of Alzheimer's disease pathophysiology, and the ability of candidate biomarkers to be measured and tracked over time. In addition to these and other important technical and scientific challenges, however, are some conceptual considerations which need to be considered, and which are the subject of this paper. It will discuss criteria for a biomarker to be used as a measure of treatment effects and some challenges in regard to each criterion. The purpose is not to discourage the very significant advances possible in the implementation of the biomarker agenda, but to lay out some of what needs to be addressed if the full value is to be realized. These criteria for whether a biomarker might be used to measure treatment effects are based on criteria for making inferences about clinical meaningfulness [4] themselves based on the Bradford-Hill criteria for establishing whether an association is causal [5]. Briefly, these criteria are: the biomarkers should, on biological grounds, plausibly be related to Alzheimer's disease; it should be statistically significant; it should show dose-dependent effects; it should show convergence with related measures; it should be replicable. Next, we consider each in a little more detail.

The biomarker should be biologically plausible. This criterion is likely to be the weakest, on three grounds. First, it will almost always be met for any biomarker, because a theoretical basis for its measurement will likely be the basis on which it is investigated in the first place. Second, as it is inherent that our understanding of biology is always contingent, and that data to the contrary will always trump a good hypothesis, no biologically plausible explanation is likely to withstand data to the contrary. Even so, the role of clinical trials in enhancing our understanding of the biology must be stressed. For example, successive failures of gamma-secretase inhibitors have given credence to the proposition that a new generation of such compounds must be selective for inhibiting a-beta in comparison with Notch signaling endpoints [6]. The ability to confirm this in human studies will help secure this understanding of the underlying biology. Even so, a particular challenge to any argument about the biological plausibility of any single candidate biomarker is this. Dementia is highly age associated. As people grow older, they are more likely to have more than one thing wrong with them, and these cumulative small effects can add up to make dementia more likely [7]; in like manner, we see in older adults, many causes of dementia existing in a single brain [8, 9]. The influence of cooccurring dementia pathologies on disease expression can pose a heavy burden on any prediction which might rest on just one biomarker. 

The association between the biomarker and disease expression should be statistically significant. On its face, this seems like a reasonable enough criterion and it might well prove to be true in practice. But what if it turns out that no single biomarker on its own will be sufficiently persuasive? This view is in fact supposed by the Dubois criteria being proposed for the diagnosis of dementia [10]. These new criteria propose several candidate biomarkers be measured, and they suggest increased predictive power will be the case when more biomarkers are present. Note, however, that a recent review suggests that most biomarkers have poor positive predictive value used on their own, and in established clinical disease are inferior to memory testing [11]. How they will work out in combination, especially to track preclinical or “pathophysiological” Alzheimer disease, when clinical symptoms are expected largely to be lacking, will be an important research question. Will it be the case that altering biomarkers of amyloid deposition will alter biomarkers of neurodegeneration to in turn alter clinical disease expression [12]? With regard to established disease, biomarkers that change as disease progresses, such as functional and metabolic markers detected by task-dependent activation on functional MRI and 18F-fluorodeoxyglucose PET, may be candidates for demonstrating statistically significant treatment effects [13]. 

The biomarker should show a dose-dependent effect. This too seems like an indubitable proposition. Not for nothing is the metaphor of “treating cholesterol” so top of mind at many biomarker discussions. Jason Karlawish of the University of Pennsylvania summed up the situation well in his presentation to the 11th International Congress of Alzheimer's Disease plenary address in Honolulu in July 2010 [14]. There he described the change in the conceptualization of Alzheimer's disease from the leading cause of dementia to a risk state. This new risk state amounts to Alzheimer's disease without dementia, which is proposed to be identified by a series of abnormalities detected by any or some combination of neuroimaging, serum, or cerebrospinal fluid abnormalities [12]. The analogy would be much like the risk of stroke, which is now indicated by an elevation in serum cholesterol. Following the biomarkers model, satisfactory treatment of Alzheimer disease would thereby be demonstrated by altering the level of the biomarker in a favourable direction, although this would be expected to hold only for biomarkers that might be expected to change with treatment—that is, those that were useful for tracking and not just diagnosis. Recent experience with an increase in hippocampal volumes in nondemented older adults who enrolled in an exercise program, compared to continued to decline to those in a sham intervention arm, is perhaps an exciting hint at how a tracking measure might work [15]. Such an effect corresponds well with established work which demonstrated that AD patients with faster cognitive deterioration tend to have greater hippocampal atrophy rates [13, 16]. Likewise, dose-dependence will itself depend on the biomarker and the stage of the patient being treated. For example, in a trial in people with established mild cognitive impairment, amyloid biomarkers might be expected largely to have plateaued whereas an impact on medial temporal atrophy might be postulated for a disease-modifying treatment. 

This hope for successful treatment of dementia being reduced to successful treatment of laboratory or imaging tests, has not gone unnoticed by the pharmaceutical and diagnosis industries. These industries have invested hundreds of millions of dollars in the “preliminary validation phase” studies of biomarkers, with the notion that the biomarkers need to be tight against clinical outcomes. But even here there is need for further questioning. If there is one theory that the traditional neuropathological studies have taught us, it is that the correlation between plaques and tangles and disease expression is modest when series include cognitively intact older adults [6, 7, 17, 18]. Whether we should expect more of contemporary biomarkers in living patients is an untested proposition. Recent experience with any amyloid-*β*_1-42_ biomarker for prodromal AD/mild cognitive impairment illustrates the problem. In detailed simulations based on estimates from the Alzheimer's Disease Neuroimaging Initiative, even though patients with prodromal AD who also had the biomarker (in their cerebrospinal fluid) shared both more impairment at baseline and more cognitive decline, they also showed more variability in outcomes [19]. In other words, the dimensionality of dementia was not reduced by the addition of biomarkers, even though this was an explicit part of the rationale for their use in clinical trials, as was the hope that treatment effectiveness could be demonstrated through biomarker change [20]. Also in pragmatic terms, this means that adding the biomarker did not improve the efficiency of the trial, in that this did not increase power or improve sample sizes. 

The criterion of convergence of measures means that if a biomarker were to successfully predict dementia, then it should be reflected in more than one dementia measure. For example, a biomarker that predicted decline in cognition as measured by the ADAS-Cog might reasonably be expected—at least in the preliminary validation phase—to predict decline in at least some of function, behaviour, quality of life, and caregiver measures. It might also expect to correlate with adverse change in more than one biomarker, on the assumption that dementia is a complex disease state. As with the other criteria, however, there might well need to be nuance in the interpretation. For example, decline in measures will reflect their time frame. If cognitive decline precedes functional impairment, following a preventive strategy, measures might not converge for some time. In addition, if a given biomarker is associated with some disease aspects more than others, convergence of measures might be modest.

As a final criterion, any result about biomarkers must be replicable. As always, this is the highest scientific standard, especially when replicated by entirely independent groups. Given the proprietary nature of much of the biomarkers enterprise, what constitutes independent replication may be a standard short of entirely independent groups. Even so, every effort should be made to achieve this standard. Usually study design consideration—double-blinding being chief amongst them—will constitute the necessary minimum.

This inventory of criteria for using biomarkers to measure treatment effects has focused on how to look. We also must consider what to look for; the recognition of Alzheimer's disease as a complex state has implications in this regard. Chief amongst these is that short of a cure, treatment for Alzheimer's disease will not be the same as having no cognitive impairment, or even age-associated or age-appropriate cognitive function. Rather, it is likely that some features of Alzheimer's disease will be present to only a trivial extent, others will be more modestly attenuated and others still might be unaffected. Whether this diversity of disease modifying effects can be captured in a single biomarker, or a battery of them, is a proposition that remains to be tested.

## References

[1] Rockwood K, Fay S, Gorman M (2010). The ADAS-cog and clinically meaningful change in the VISTA clinical trial of galantamine for Alzheimer’s disease. *International Journal of Geriatric Psychiatry*.

[2] Robert P, Ferris S, Gauthier S, Ihl R, Winblad B, Tennigkeit F (2010). Review of Alzheimer’s disease scales: is there a need for a new multi-domain scale for therapy evaluation in medical practice?. *Alzheimer’s Research and Therapy*.

[3] Atkinson  AJ, Colburn WA, DeGruttola VG (2001). Biomarkers and surrogate endpoints: preferred definitions and conceptual framework. *Clinical Pharmacology and Therapeutics*.

[4] Rockwood K, Macknight C (2001). Assessing the clinical importance of statistically significant improvement in anti-dementia drug trials. *Neuroepidemiology*.

[5] Hill AB (1984). *A Short Textbook of Medical Statistics*.

[6] Basi GS, Hemphill S, Brigham EF (2010). Amyloid precursor protein selective gamma-secretase inhibitors for treatment of Alzheimer’s disease. *Alzheimer’s Research &amp; Therapy*.

[7] Song X, Mitnitski A, Rockwood K (2011). Non-traditional risk factors combine to predict Alzheimer’s disease and dementia. *Neurology*.

[8] Savva GM, Wharton SB, Ince PG, Forster G, Matthews FE, Brayne C (2009). Age, neuropathology, and dementia. *The New England Journal of Medicine*.

[9] White L (2009). Brain lesions at autopsy in older japanese-american men as related to cognitive impairment and dementia in the final years of life: a summary report from the honolulu-asia aging study. *Journal of Alzheimer’s Disease*.

[10] Dubois B, Feldman HH, Jacova C (2007). Research criteria for the diagnosis of Alzheimer’s disease: revising the NINCDS-ADRDA criteria. *The Lancet Neurology*.

[11] Gomar JJ, Bobes-Bascaran MT, Conejero-Goldberg C, Davies P, Goldberg TE (2011). Utility of combinations of biomarkers, cognitive markers, and risk factors to predict conversion from mild cognitive impairment to Alzheimer disease in patients in the Alzheimer's disease neuroimaging initiative. *Archives of General Psychiatry*.

[12] Sperling RA, Aisen PS, Beckett LA (2011). Toward defining the preclinical stages of Alzheimer's disease: recommendations from the National Institute on Aging-Alzheimer's Association workgroups on diagnostic guidelines for Alzheimer's disease. *Alzheimer's and Dementia*.

[13] Frisoni GB, Fox NC, Jack CR, Scheltens P, Thompson PM (2010). The clinical use of structural MRI in Alzheimer disease. *Nature Reviews Neurology*.

[14] Karlawish J (2010). The meaning of Alzheimer’s disease across time and place. *Alzheimer’s and Dementia*.

[15] Erickson KI, Voss MW, Prakash RS (2011). Exercise training increases size of hippocampus and improves memory. *Proceedings of the National Academy of Sciences of the United States of America*.

[16] Barnes J, Ourselin S, Fox NC (2009). Clinical application of measurement of hippocampal atrophy in degenerative dementias. *Hippocampus*.

[17] Imhof A, Kövari E, von Gunten A (2007). Morphological substrates of cognitive decline in nonagenarians and centenarians: a new paradigm?. *Journal of the Neurological Sciences*.

[18] Bennett DA, Schneider JA, Bienias JL, Evans DA, Wilson RS (2005). Mild cognitive impairment is related to Alzheimer disease pathology and cerebral infarctions. *Neurology*.

[19] Schneider LS, Kennedy RE, Cutter GR (2010). Requiring an amyloid-*β*1-42 biomarker for prodromal Alzheimer’s disease or mild cognitive impairment does not lead to more efficient clinical trials. *Alzheimer’s and Dementia*.

[20] Shaw LM, Vanderstichele H, Knapik-Czajka M (2009). Cerebrospinal fluid biomarker signature in alzheimer's disease neuroimaging initiative subjects. *Annals of Neurology*.

